# Multiple Comorbid Conditions among Middle-Aged and Elderly Hemophilia Patients: Prevalence Estimates and Implications for Future Care

**DOI:** 10.4061/2011/985703

**Published:** 2011-09-07

**Authors:** Aroub A. Khleif, Nidra Rodriguez, Deborah Brown, Miguel A. Escobar

**Affiliations:** ^1^Department of Pediatrics and Gulf States Hemophilia & Thrombophilia Center, The University of Texas Health Science Center at Houston, Houston, TX 77030, USA; ^2^Department of Internal Medicine and Gulf States Hemophilia & Thrombophilia Center, The University of Texas Health Science Center at Houston, Houston, TX 77030, USA

## Abstract

*Introduction*. Advances in hemophilia care and treatment have led to increases in the life expectancy among hemophiliacs. As a result, persons with hemophilia are reaching an older age and experiencing various age-related health conditions never seen before in this population. *Aim*. To determine the prevalence of comorbidities among middle-aged and elderly hemophilia A and hemophilia B patients. *Methods*. Retrospective chart review among all hemophilia patients, who attended the Gulf States Hemophilia and Thrombophilia Center. *Results*. All patients had at least one comorbid condition other than hemophilia, and the majority had between 3 and 6 comorbidities. The most common conditions identified were chronic hepatitis C, hypertension, HIV, chronic arthropathy, and overweight/obesity. *Conclusions*. Since persons with comorbidities are more likely to have poorer health outcomes and require greater care in managing their health needs, caring for aging hemophiliacs is likely to pose various social and economic challenges for both patients and providers.

## 1. Introduction

During the past three decades, the life expectancy for individuals with hemophilia A (a hereditary deficiency of coagulation factor VIII) and hemophilia B (a hereditary deficiency of coagulation factor IX) has markedly increased, primarily due to advances in medical care, as well as the introduction and availability of clotting factor replacement products and effective treatments for infectious diseases [1–10]. However, with this increased longevity and improved quality of life, comes a generation of middle-aged and elderly hemophiliacs that are experiencing age-related health conditions that have subsequently not been seen in this population [1, 6, 10]. 

Prior to the availability of factor replacement therapy, the majority of patients with hemophilia died at an early age from bleeding problems [2, 4, 11]. Throughout the 1980s and 1990s, high death rates were observed, due to blood-borne viral infections, specifically human immunodeficiency virus (HIV) and the hepatitis C virus (HCV), and replaced bleeding complications as the primary cause of death among hemophiliacs [5, 12]. Nevertheless, barring the increased mortality rates discerned during this period, the life expectancy for patients with hemophilia, particularly those with mild or moderate hemophilia, in high and middle income nations, is currently drawing near to that of the general male population [13, 14].

While a variety of age-related co-morbidities have been reported among males in the general population, only a modest amount of information exists regarding these new causes of age-related morbidity and mortality and more specifically how to deal with these conditions among individuals with hemophilia [1, 5, 6, 11]. The primary focus of this study was to identify and estimate the prevalence of co-morbid conditions that were associated with middle-aged and elderly hemophilia patients attending at Texas treatment center and evaluate the implications these conditions may have on future care among individuals in this population.

## 2. Materials and Methods

We conducted a retrospective study among adult patients, 40 years of age or greater, who had a clinical diagnosis of hemophilia A or hemophilia B, and attended the Gulf States Hemophilia and Thrombophilia Center (GSHTC) at least once between January 2007 and August 2010. The GSHTC is the largest treatment center in the southwest and one of the largest in the country. 

Data were obtained from the patient's medical charts using a standardized data abstraction form, and information regarding the patient's demographic characteristics, as well as clinical and co-morbid conditions, was examined. Laboratory findings, in conjunction with physician notes as well as medication and problem lists from the patient's medical charts, were used to determine whether or not they had a specific comorbidity. Furthermore, the diagnostic criteria used to define individual co-morbid conditions are described in Table 1. Patients who met the inclusion criteria but died during the specified observation range were also included in the study. In addition, we defined comorbidity as the presence of one or more diseases/conditions, other than the patient's hemophilia disease. 

Frequency distributions of demographic and treatment-related characteristics by hemophilia disease were examined. Differences between patients with hemophilia A and hemophilia B were assessed using Fischer's exact test. In addition, the prevalence of co-morbid conditions was examined among this patient cohort and compared to that of general US male population when data were available. All analyses were conducted using STATA statistical analysis software, version 11.0 [15].

## 3. Results

Characteristics of the study sample according to their disease status are summarized in Table 2. Between January 2007 and August 2010, the GSHTC saw a total of 404 hemophilia patients, roughly 16% were 40 years of age or greater. Of the 63 patients who met the inclusion criteria, the majority had hemophilia A, 71%, respectively. The mean age of the cohort was approximately 53 years for patients with hemophilia A and 54 years among patients with hemophilia B. In addition, the majority of the sample visited the treatment center within one year of the observation period was treated on demand and had mild hemophilia. Patients with hemophilia A and hemophilia B did not differ significantly on any of the variables examined with the exception of race/ethnicity and employment status.

Table 3 presents the prevalence of specific co-morbidities among the study sample, including comparisons to the age-matched, general US male population when data were available. Confidence limits were also estimated in order to draw comparisons to that of the general US population. However, due to the small sample size of the study cohort, it is difficult to draw significant comparisons of the representativeness of the prevalence estimates reported between our patient population and that of the general US male population. All patients in the study had at least one co-morbid condition other than their hemophilia disease, with the majority having between 3 and 6 conditions (mean number of conditions in the sample was 4). As expected, more than half of the sample suffered from hemophilic arthropathy, 55% of which had severe hemophilia. Only 21% of the cohort (13 patients) did not have any blood-borne viral infections: 6% had hepatitis B virus (HBV) infection, 25% had HIV, 78% had HCV, 25% were coinfected with HIV and HCV, and 5% had both HBV and HCV. Additionally, differences in co-morbidities between hemophilia A and hemophilia B patients were also examined. While the results did show differences between the groups with respect to their HIV, HCV, cardiovascular disease, renal disease, and arthropathy status, it is difficult to provide any meaningful statistically significant comparisons due to the small sample size of the study cohort. 

A total of 5 patients (8%) died during the study period. Causes of death included hepatocellular carcinoma (HCC), acute myeloid leukemia, and lung cancer, and 2 patients died from accidental, nonmedical factors. Additionally, 5 patients in the sample, all of whom had hemophilia A (1 patient had mild hemophilia A, and 4 patients had severe hemophilia A), received a liver transplant during the observation period. Among patients receiving a liver transplant, 80% were diagnosed with end-stage liver disease due to HCV. 

Over three-fourths (78%) of the study population had at least one cardiovascular risk factor including hypertension (46%), a body mass index (BMI) of 25.0 or higher (65% of the sample was overweight or obese), high cholesterol (16%), and diabetes mellitus (14%). In addition, the prevalence of other co-morbidities that were examined among patients in this population are reported in Table 2 and include renal disease (8%), thyroid disease (6%), depression (8%), neurological disease (5%) including Parkinson's disease, epilepsy, and dementia, respiratory disease (11%) including asthma, sleep apnea, sarcoidosis, and chronic obstructive pulmonary disease, and gastrointestinal disease (5%) including peptic ulcer disease and acid reflux disease.

## 4. Discussion

This study revealed a variety of notable differences with regard to comparing the prevalence of multiple co-morbid conditions among middle-aged and elderly hemophilia A and hemophilia B patients to that of the general US male population. Previous research has suggested that as much as 62% of the US population over the age of 65 have more than one co-morbid condition [21]. Results from this study indicated that all of the patients in this sample had more than one co-morbid condition other than their underlying hemophilia disease, and roughly 19% of the cohort over the age of 65 had at minimum 3 or more co-morbid conditions. 

As noted in previous studies, the prevalence of HIV and HCV infections are considerably higher among hemophiliacs than in the general male population [4, 5] and are a leading cause of morbidity and mortality in this population [2, 22, 23]. It is estimated that greater than 90% of hemophiliacs who were treated with plasma-derived factor concentrates before 1985 became infected with HCV, and greater than 55% of these patients were also coinfected with HIV [24]. Among patients in this cohort who were between 40 and 49 years of age (*N* = 29), the prevalence of HIV infection was approximately 38% compared to 0.74% among age-matched males in the US. In addition, the prevalence of HCV infection in our patient sample was 12 to 90 times higher than in the age-matched male population (40–49 years: 72% versus 6%; 50–59 years: 83% versus 1.6%; ≥60 years: 81% versus 0.9%). Furthermore, chronic HCV infection is a major risk factor in the development of liver cirrhosis and HCC [11, 25], and, subsequently, HCC has emerged as a significant cause of mortality among HCV-infected hemophiliacs [11]. Roughly 11% of patients in this sample who were diagnosed with HCV progressed to HCC; one patient died, and more than half (57%) received a liver transplant. Since the risk of HCC among HCV-infected hemophilia patients increases with older age and the presence of HIV infection [11, 22, 23], it is expected that many of the HCV-infected patients in this sample will progress to end-stage liver disease or HCC. 

While cardiovascular disease (CVD) has been the primary cause of death among individuals in the US for the past eight decades [26], several studies have reported that mortality from cardiac events among individuals with hemophilia is lower than in the general population [7, 8, 13, 27, 28] and hemophilia may actually have a protective effect against CVD [4, 11, 28]. The prevalence of CVD among patients between 40 and 59 years of age in this study was similar compared to the age-matched US male population (40% versus 39%), but slightly lower among those patients of 60 years of age or greater (69% versus 71%). However, it should be noted that in order to provide comparisons between this cohort to that of age-matched males in the US, patients with CVD were defined as having any of the following conditions (based on the National Center for Health Statistics definition): congestive heart disease, heart failure, stroke, and hypertension. If we excluded hypertension from the analysis and defined CVD as having only one of the following conditions: congestive heart disease, heart failure and/or stroke, the prevalence of CVD among all patients in the study was approximately 11%.

Despite conflicting reports regarding a possible protective effect against CVD among individuals with hemophilia [4, 11, 28], common risk factors for CVD including hypertension, high cholesterol, diabetes mellitus (DM), and overweight/obesity were observed among patients in this study. The prevalence of hypertension was lower compared to the US population among all age groups except among patients between 55 and 64 years of age. No differences were observed between our patient population and the age-matched general population with respect to high cholesterol. While over 65% of this cohort was overweight or obese, these rates were lower compared to the age-matched population. In addition, overweight and obesity were more prevalent among patients with mild hemophilia (72%), which is similar to a finding reported in a previous study [29]. 

An increased bodyweight is an important risk factor associated not only with CVD, but also in the development of DM and chronic arthropathy. Only a limited amount of information exists regarding the prevalence of DM among hemophiliacs. The study conducted by Walsh et al. [30] estimated that the prevalence of DM among mild hemophiliacs was 24% compared to 6% in control males (mean age of subjects in both groups was 46 years of age) [30]. The prevalence of DM among all patients in this sample was 14%, and roughly 89% of those patients who had DM also had a BMI greater than 25. 

The majority of hemophiliacs born prior to the availability of prophylactic therapy suffer from hemophilic arthropathy [4], which continues to be the primary cause of morbidity among individuals in this population [2]. As expected, half of the patients in this cohort suffer from chronic arthropathy, of which 56% had severe hemophilia and 53% had a BMI of 25 or higher. In addition, overweight and obesity may have greater implications on persons with hemophilia due to the fact that an increased bodyweight may cause additional damage to already deteriorated joints [29]. Furthermore, since the dosage of factor replacement treatment is based on bodyweight, the costs of care are much higher among overweight and obese patients compared with patients with a normal bodyweight. 

Renal disease is another age-related medical condition that affects hemophiliacs, who are reported to have up to a 50-fold increase in mortality due to renal failure compared to the general population [31]. Previous research has indicated that risk factors for renal failure among individuals with hemophilia include increased age, hypertension, and HIV co-infection [1, 32]. Approximately 8% of patients in this sample had chronic renal disease, 20% of which also had HIV, and 40% of which had hypertension. With the mean age of all patients in this cohort being 53 years of age, it is likely that more cases of renal disease will be observed as the population continues to age.

While hemophilia care has undergone substantial advancements during the past three decades, a variety of healthcare needs arising from many of the age-related co-morbidities mentioned in this paper pose significant challenges for the treatment and management of these conditions among aging hemophiliacs. Currently, there are few evidence-based guidelines that direct medical professionals on how to most effectively manage the comprehensive care needs of hemophilia patients with these age-related co-morbidities [1, 11]. As a result, optimal treatment and care for hemophiliacs with multiple co-morbid conditions has been challenging for both healthcare providers and the patient.

Further studies are needed to document the safety and efficacy of certain drug therapies, procedures, and lifestyle changes among aging hemophiliacs with multiple co-morbid conditions. For example, no explicit guidelines are available for addressing CVD risk in persons with hemophilia. While there are modifiable lifestyle factors that can be addressed including diet, exercise, and smoking cessation programs [11], safety of frequent aspirin use needs to be evaluated as this could cause an increase in the bleeding frequency among hemophiliacs. In addition, with regard to lifestyle factors such as exercise and activity programs to help reduce the risk of CVD as well as other conditions such as overweight/obesity and diabetes, these may not be practical and/or may be difficult particularly for patients with chronic arthropathy. Support from physical therapists at treatment centers may play a critical role in promoting an active lifestyle within the boundaries of a patient's abilities. 

Given the complexities associated with how to best care for and treat hemophiliacs with multiple co-morbid conditions, successful management must entail a multifaceted approach, which encompasses drawing from expertise not only from hematology, but that of cardiology, oncology, urology, infectious disease, orthopedics, hepatology, nephrology, and internal medicine. A lack of coordination between these various departments may affect the delivery of appropriate and timely healthcare services. Hemophilia treatment centers should play an essential role in coordinating the care for these patients to ensure they are knowledgeable of the services they need as well as understand the implications associated with seeking timely care from these services. However, management and treatment requirements for specific co-morbidities need to be an interdisciplinary effort, and hemophilia treatment centers cannot be the sole caregivers for hemophiliacs with multiple co-morbidities. As such, lifestyle issues as well as general screening programs should be incorporated in the management and treatment plans, including assistance with timely referrals and followup with appropriate specialty services for aging hemophilia patients attending treatment centers.

## 5. Conclusions

Individuals with multiple co-morbid conditions tend to receive suboptimal care, which can lead to poorer health outcomes and increased treatment costs. In addition, the challenge of providing adequate care increases in complexity as the number of chronic conditions increases [21, 33], resulting in disproportionately high health care costs. It is estimated that over 75% of health care expenditures in the US are spent on care for persons with multiple co-morbidities [21] and the average spending per person with co-morbidities is roughly five times greater compared with individuals with no chronic conditions [33]. As the world population of persons with hemophilia ages, an increase in age-related health conditions not previously seen in this population are likely to become more prevalent. Hemophilia is an already costly disease, and understanding both treatment care needs and its related costs among aging hemophiliacs with multiple co-morbidities is critical in order to provide optimal care and manage their comprehensive health needs effectively. While this study identified a variety of co-morbidities associated with middle-aged and elderly hemophiliacs, only 63 patients were included in the cohort, thus making it difficult to provide any statistically significant comparisons. Moreover, due to the small sample size of the cohort, our estimates of co-morbidities make it difficult to draw significant comparisons between our hemophilia patient population and the general US male population. In addition, since patients were classified as having a specific condition based on information available in their medical charts, the possibility of underestimating certain co-morbidities may be of concern. Further research, particularly larger, multicenter, prospective studies are needed to identify how to best care for and treat aging hemophilia patients with multiple co-morbidities.

## Figures and Tables

**Table 1:** Diagnostic criteria for defining co-morbid conditions.

Condition	Criteria^‡^
Human immunodeficiency virus	HIV-1 PCR quantification
	On drug therapy (HAART)

Hepatitis C virus	HCV RNA
	On or received drug therapy

Overweight/obesity	Based on their BMI^†^ score, patients were categorized as overweight or obese based on the following:
	Overweight: 25.0–29.9
	Obesity: 30.0 and above

Cardiovascular disease	Echocardiogram findings
	Electrocardiogram findings
	On drug therapy
	Cardiologist notes

Hypertension	≥130 mmHg systolic blood pressure
	≥85 mmHg diastolic blood pressure
	Primary care physician notes
	On drug therapy for elevated blood pressure

Hypercholesterolemia	Lipid profile
	On drug therapy for high lipids
	Primary care physician notes

Chronic arthropathy	Physical findings
	Radiographic findings

Hepatitis B virus	Hepatitis B surface antigen detected for more than 6 months

Diabetes	Primary care physician notes
	On drug therapy

Renal disease	Patient is on dialysis
	Pathology results from biopsy
	Estimation of glomerular filtration rate (GFR)
	Nephrologist notes

Depression	On drug therapy
	Psychiatry notes

Respiratory diseases	Positive radiographic findings
	Primary care physician notes
	On drug therapy or continuous positive airway pressure (CPAP) therapy

Gastrointestinal diseases	Gastroenterologist notes
	Endoscopy findings
	On drug therapy

Thyroid disease	On drug therapy

Neurological disease	Neurologist or primary care physician notes
	On drug therapy

Cancer	Oncologist notes
	Pathology reports
	Radiographic findings

^‡^Patient was considered to have a specific comorbidity if any of the criteria for that condition was indicated in their chart.

^†^National Center for Health Statistics. Health, United States, 2009, Hyattsville, MD, 2010.

**Table 2:** Selected characteristics of patients 40 years of age or greater, who visited the Gulf States Hemophilia & Thrombophilia Center between January 1, 2007 and August 31, 2010, according to disease status.

Characteristics	Hemophilia A (*N* = 45)		Hemophilia B (*N* = 18)		Total (*N* = 63)		*P* value^†^
	*n*	%	*n*	%	*N*	%	
*All patients*		71.4		28.6			
Age in years							0.715
40–49	21	46.6	8	44.5	29	46.0	
50–59	14	31.1	4	22.2	18	28.6	
60–69	7	15.6	4	22.2	11	17.5	
≥70	3	6.7	2	11.1	5	7.9	

Race/ethnicity							0.002
Caucasian (non-Hispanic)	26	57.8	8	44.4	34	54.0	
Hispanic	16	35.5	2	11.2	18	28.6	
African American	3	6.7	8	44.4	11	17.4	

Employment status							0.032
Employed full-time	18	40.0	3	16.6	21	33.4	
Employed part-time	5	11.1	0	—	5	7.9	
Unemployed	22	48.9	14	77.8	36	57.1	
Unknown	0	—	1	5.6	1	1.6	

Insurance status							0.283
Commercial insurance	18	40.0	8	44.4	26	41.3	
Medicare	13	28.9	9	50.0	22	34.9	
Medicaid	5	11.1	0	—	5	7.9	
State high risk	1	2.2	0	—	1	1.6	
Uninsured	8	17.8	1	5.6	9	14.3	

Disease severity							0.694
Mild (6%–50%)	23	51.1	9	50.0	32	50.8	
Moderate (1%–5%)	6	13.3	4	22.2	10	15.9	
Severe (<1%)	16	35.6	5	27.8	21	33.3	

Treatment type							0.248
On demand	35	77.8	15	83.3	50	79.4	
Intermittent prophylaxis	0	—	1	5.6	1	1.6	
Continuous prophylaxis	10	22.2	2	11.1	12	19.0	

Last visit to treatment center							0.201
<1 year ago	30	66.7	13	72.2	43	68.3	
1-2 years ago	5	11.1	4	22.2	9	14.3	
>2 years ago	10	22.2	1	5.6	11	17.4	

^†^Due to small sample sizes, *P* value for Fischer's Exact Test is reported.

**Table 3:** Prevalence estimates and 95% confidence intervals of co-morbid conditions among hemophilia patients compared to the general US male population, by selected characteristics.

Condition	Hemophilia A and B patients (*N* = 63)			Prevalence in general US male population^†^
	*n*	%	95% CI^‡^	%
Total number of co-morbid conditions^††^				
1-2	16	25.4	14.35, 36.45	
3-4	22	34.9	22.82, 47.02	
5-6	17	27.0	15.72, 38.25	
≥7	8	12.7	4.24, 21.15	

HIV positive	16	25.4	14.35, 36.45	^a^40–49 years: 0.74
40–49 years	11	37.9	19.15, 56.71	

HCV positive	49	77.8	67.22, 88.33	^b^40–49 years: ~ 6 50–59: 1.6 ≥60 years: 0.9
40–49 years	21	72.4	55.11, 89.72	
50–59 years	15	83.3	64.26, 99.02	
≥60 years	13	81.3	59.77, 99.03	

BMI				
Normal (18.5–24.9)	22	34.9	22.82, 47.02	
Overweight (25.0–29.9)	25	39.7	27.26, 52.10	
Obese (≥30)	16	25.4	14.34, 36.45	

BMI ≥25.0				^c^BMI≥25.0 40–59 years: 77.8 ≥60 years: 78.4
40–59 years	33	70.2	56.64, 83.79	
≥60 years	8	50.0	22.48, 77.52	

BMI ≥30.0				^c^BMI≥30.0 40–59 years: 34.3 ≥60 years: 37.1
40–59 years	13	27.7	14.38, 40.93	
≥60 years	3	18.8	—	

BMI ≥35.0				^c^BMI≥35.0 40–59 years: 11.6 ≥60 years: 11.6
40–59 years	6	12.8	2.8, 22.67	
≥60 years	1	6.3	—	

BMI ≥40.0				^c^BMI≥40.0 40–59 years: 4.2 ≥60 years: 4.2
40–59 years	2	4.3	—	
≥60 years	—	—	—	

Cardiovascular disease^†††^	30	47.6	34.94, 60.30	^d^40–59 years: 39.1 ≥60 years: 71.3
40–59 years	19	40.4	25.86, 54.99	
≥60 years	11	68.8	43.24, 94.26	

Hypertension	29	46.0	33.38, 58.69	
40–44 years	6	46.2	14.80, 77.51	^e^45–54 years: 36.2 55–64 years: 50.2 65–74 years: 64.1 ≥75 years: 65.0
45–54 years	7	30.4	10.09, 50.78	
55–64 years	8	53.3	24.74, 81.93	
65–74 years	6	60.0	23.06, 96.94	
≥75 years	2	100	—	

Hypercholesterolemia	10	15.9	6.60, 25.15	
45–54 years	4	17.4	0.63, 34.15	^e^45–54 years: 36.2 55–64 years: 50.2 65–74 years: 64.1 ≥75 years: 65.0
55–64 years	2	13.3	—	
65–74 years	2	20.0	—	
≥75 years	1	50.0	—	

Chronic arthropathy	33	52.4	39.70, 65.06	
40–59 years	22	46.8	32.00, 61.62	
≥60 years	11	68.8	43.24, 94.26	
HBV positive	4	6.4	0.16, 12.54	

Diabetes	9	14.3	5.40, 23.17	

Renal disease	5	7.9	1.07, 14.80	

Depression	5	7.9	1.07, 14.80	

Respiratory disease	7	11.1	3.13, 19.09	

Gastrointestinal disease	3	4.8	—	

Thyroid disease	4	6.4	0.16, 12.54	

Neurological disease	3	4.8	—	

Cancer	10	15.9	6.60, 25.15	
Hepatocellular carcinoma	7	70.0		
Non-Hodgkin's lymphoma	1	10.0		
Lung cancer	1	10.0		
Leukemia	1	10.0		

^‡^CI: Confidence interval,

^†^Prevalence estimates reported when data were available:

^a^see [16].

^b^see [17].

^c^see [18].

^d^see [19].

^e^see [20].

^††^Number of conditions excluding hemophilia disease.

^†††^Includes patients with cardiovascular disease, hypertension, and stroke.
